# Suspected intracranial hypertension in COVID-19 patients with severe respiratory failure

**DOI:** 10.1371/journal.pone.0310077

**Published:** 2024-09-19

**Authors:** Marco Carbonara, Erica Ferrari, Tatiana Birg, Veronica Punzi, Francesca Bichi, Beatrice Lazzari, Valentina Palmaverdi, Nicola Bottino, Fabrizio Ortolano, Tommaso Zoerle, Giorgio Conte, Nino Stocchetti, Elisa R. Zanier, The UOC Terapia Intensiva Presidio Temporaneo Fiera Milano investigators group

**Affiliations:** 1 Department of Anesthesia and Critical Care, Neuroscience Intensive Care Unit, Fondazione IRCCS Ca’ Granda Ospedale Maggiore Policlinico, Milan, Italy; 2 University of Milan, Milan, Italy; 3 Department of Anesthesia and Critical Care, General Intensive Care Unit, Fondazione IRCCS Ca’ Granda Ospedale Maggiore Policlinico, Milan, Italy; 4 Department of Neuroradiology, Fondazione IRCCS Ca’ Granda Ospedale Maggiore Policlinico, Milan, Italy; 5 Department of Neuroscience, Istituto di Ricerche Farmacologiche Mario Negri IRCCS, Milan, Italy; Universitatsklinikum Regensburg, GERMANY

## Abstract

**Background:**

COVID-19 patients may exhibit neurological symptoms due to direct viral damage, systemic inflammatory syndrome, or treatment side effects. Mechanical ventilation in patients with severe respiratory failure often requires sedation and neuromuscular blockade, hindering thorough clinical examinations. This study aimed to investigate neurological involvement through clinical and noninvasive techniques and to detect signs of intracranial hypertension in these patients.

**Method:**

We conducted a prospective observational study on mechanically ventilated COVID-19 adult patients admitted to our ICU, following standard of care protocols for ventilation and permissive hypercapnia. Data were collected at three time points: admission day (T1), day seven (T7), and day fourteen (T14). At each time point, patients underwent multimodal noninvasive neurological monitoring, including clinical examination, pupillary reactivity, transcranial color doppler of the middle cerebral artery (MCA), and optic nerve sheath diameter (ONSD) assessed via ultrasound (US). Head computer tomography (CT) was performed at T1 and T14. A limited subset of patients had a follow-up examination six months after ICU discharge.

**Results:**

Seventy-nine patients were recruited; most were under deep sedation and neuromuscular blockade at T1. Pupillary size, symmetry, and reactivity were normal, as was the MCA mean velocity. However, ONSD, assessed by both US and CT, appeared enlarged, suggesting raised intracranial pressure (ICP). In a subgroup of 12 patients, increased minute ventilation was associated with a significant decrease in US-ONSD, corresponding to a drop in paCO2. At follow-up, twelve patients showed no long-term neurological sequelae, and US-ONSD was decreased in all of them.

**Discussion and conclusions:**

In this cohort, enlarged ONSD was detected during non-invasive neurological monitoring, suggesting a raised ICP, with hypercapnia playing a prominent role. Further studies are needed to explore ONSD behavior in other samples of mechanically ventilated, hypercapnic patients.

## Background

SARS-CoV-2 infection can affect various organs, such as the lungs, the vascular system, and the kidneys. The central nervous system (CNS) [1–3] is often involved due to direct viral damage or systemic inflammatory syndrome. The severity of neurological manifestations varies, ranging from subjective symptoms (ageusia and anosmia) to overt neuropsychiatric alterations and cerebrovascular events [4, 5]. Additionally, the CNS may be affected by the side effects of common treatment strategies, such as anti-coagulation [6], which can cause intracranial bleeding and high positive end-expiratory pressure (PEEP) [7, 8] and prone positioning [9, 10], which may affect venous return from the brain and contribute to intracranial hypertension.

Neurological examination becomes particularly difficult when the severity of respiratory failure calls for artificial ventilation and prone positioning. Continuous deep sedation and intravenous neuromuscular blocking agents are routine in these cases. Classical invasive neuro-monitoring techniques, such as intracranial pressure (ICP), brain tissue oxygen tension, or microdialysis, are unsuitable because placing probes in the brain tissue is contraindicated in patients receiving anticoagulants.

Due to these constraints, we applied a ‘bundle’ of CNS exploration, combining repeated non-invasive measurements (clinical examination, pupillometry, optic nerve sheath diameter (ONSD), and transcranial color Doppler (TCCD)) with sequential CT scan imaging in mechanically ventilated patients with severe SARS-CoV-2 respiratory failure admitted to ICUs. The aims of this study were:

1. to investigate the neurological involvement in the first two weeks following ICU admission;
2. to detect signs of intracranial hypertension.

## Methods

The study was approved by the local Ethics Committee of the Fondazione IRCCS Ca’ Granda Ospedale Maggiore Policlinico, Milan (approval #868_2020, 28.10.2020). Informed consent for incapacitated cases was obtained in two steps: first, information was given to the next of kin, who were verbally asked for preliminary approval; the written consent form was submitted directly to the patient once they recovered.

The study was designed as non-pharmacological, longitudinal, and observational. We included consecutive patients between November 2, 2020, and February 28, 2021, with the following inclusion criteria: invasive mechanical ventilation in COVID-19-related respiratory failure, confirmed by real-time polymerase chain reaction, and ICU admission.

We excluded patients under the age of 18, patients with pre-existing neurological diseases, and pregnant women.

Patients were admitted to the “Hospital in the Fair” a temporary emergency extension of the Milan Policlinico University Hospital, built to cope with the COVID-19 pandemic. This hospital was designed as a comprehensive ICU structure where seven ICU modules (14–16 ICU beds each) cooperated in offering intensive care to patients with severe respiratory failure who could not be treated in a “regular” hospital overwhelmed by the number of admissions. Clinical management (including mechanical ventilation settings and pharmacological therapies) followed the Institutional standard of care for COVID-19 related respiratory failure [11]. Briefly, PEEP was titrated, and rescue oxygenation maneuvers (such as prone position and nitric oxide) were used, depending on the clinical severity. Hemodynamic, respiratory, and metabolic stability were ensured, including correcting any hypotensive state with fluids and vasopressors. Sedation was provided by continuous infusion of propofol and midazolam, analgesia by fentanyl or remifentanil, and muscle relaxants such as rocuronium were administered to improve patient-ventilator synchrony and when prone position became necessary. All patients received corticosteroids per the Recovery trial [12] and were fully anti-coagulated with a therapeutic dose of low molecular weight heparin (100 units/kg twice daily).

Patients were screened for the study and enrolled on the day of ICU admission. Data were collected at three time points during the ICU stay: admission day (T1), day seven (T7), and day fourteen (T14). A limited subset of patients received a follow-up neurological examination, and the ONSD was measured again by ultrasound (US) at six months after ICU discharge.

The multimodal neurological monitoring included

- *Level of consciousness and sedation*
- *Pupils and pupillometry*

All patients had clinical examinations to assess pupil size and reactivity. When a pupillometer (Neuroptics, Irvine, USA) was available, the size, reactivity to light, and NPi, a proprietary index quantifying the pupillary reflex to light and its velocity, were measured. NPi is computed in arbitrary units, with values greater than 3 indicating a physiological response [13].

- *Optic nerve sheath diameter (ONSD)*

The point-of-care ultrasound GE Healthcare LOGIQ-e plane transducer 12L-RS (1.7–2 Hz) ultrasound probe was gently laid on the patient’s closed eyelid, interposing a transparent plastic film and a layer of ultrasound gel. When the optic nerve sheath was identified, the diameter was measured 3 mm from the posterior globe margin as the space between the hyperechoic external borders of the nerve, perpendicularly to its longitudinal axis. The ONSD was obtained by averaging six consecutive measures for each eye, three recorded in the transverse plane and three in the sagittal plane, respecting the eye bulb. In some cases, clinically necessary minute ventilation adjustments caused a change in arterial partial pressure of carbon dioxide (paCO2). This enabled us to test the US-ONSD response to different levels of paCO2 dynamically.

- *Transcranial color Doppler* (TCCD)

The wide-band phased array 6S-RS (2.5–7.0 MHz) of the GE Healthcare LOGIQ-e ultrasound machine was used to investigate the middle cerebral artery (MCA) using the temporal window bilaterally whenever possible.

The MCA point with the highest velocity was chosen at every time point for each patient, depending on the available ultrasound window.

- *Brain CT scan*

CT images were acquired using a 64-slice CT scanner (Ingenuity, Philips Healthcare, Netherlands). The head scanning protocol included 0.6-mm axial, non-overlapping contiguous sections. Bone and brain algorithm reconstructions were available for review. The image processing and analysis were done on a PACS workstation (Synapse, Fujifilm, Japan). Two experienced neuroradiologists (GC and MF) obtained multiplanar reconstructions to assess the ONSD bilaterally, 3 mm behind the optic globe in the axial and coronal planes (i.e., respectively parallel and perpendicular to the axis of the optic nerves). The mean of the axial and coronal measurements was calculated for each eye. The two operators’ average measurements were used for analysis. All measurements were taken at constant window level and width settings (80 and 400 HU, respectively). When possible, the ONSD was checked on US on the same day as the CT scan. Neuroradiologists also reported all possible signs of intracranial hypertension, scoring the extent of sulcal effacement, peri mesencephalic cistern effacement or disappearance, and loss of distinction between gray and white matter [14–16]. The duration of invasive mechanical ventilation, ICU length of stay (LOS), and ICU mortality were recorded for all cases.

A six-month follow-up was obtained in a subset of 12 patients, providing information about long-term neurological status and US-ONSD. Venous blood gas analysis was performed on these patients, and samples from basilic or median cubital veins were collected to define the current respiratory status. The arterial CO_2_ level corresponding to the peripheral venous CO_2_ tension was calculated as in Eq (1):

$$arterialCO_{2}=peripheralvenousCO_{2}-4.8$$ (1)

with reference to the results reported by Zeserson and colleagues [17].

### Statistical analysis

The neurological parameters were followed over time, and their relationships with other clinical variables are described here. Continuous variables are presented as median and interquartile range (IQR). Categorical variables are expressed as absolute frequencies and percentages. For continuous variables, the differences between time points are represented by boxplots. A spaghetti plot [18–20] was used to graphically represent the relations between PaCO_2_ and ONSD in each subject.

A linear regression model was implemented with the patient as a random effect, MCA as an outcome, and time as a covariate to assess whether the MCA pattern was significant over time. The association between PaCO_2_ and ONSD was tested with a linear regression model. The Wilcoxon test was used to assess ONSD differences before and after the adjustment in minute ventilation, or comparing measurements taken six months after the patient’s discharge with the last one available during hospitalization. The agreement between the US and CT ONSD measurements was described using a Bland and Altman plot.

All statistical tests were two-tailed, and p-values less than 0.05 were considered significant.

All analyses used R statistical software (Foundation for Statistical Computing, Vienna, Austria).

## Results

### General data

**Fig 1:**
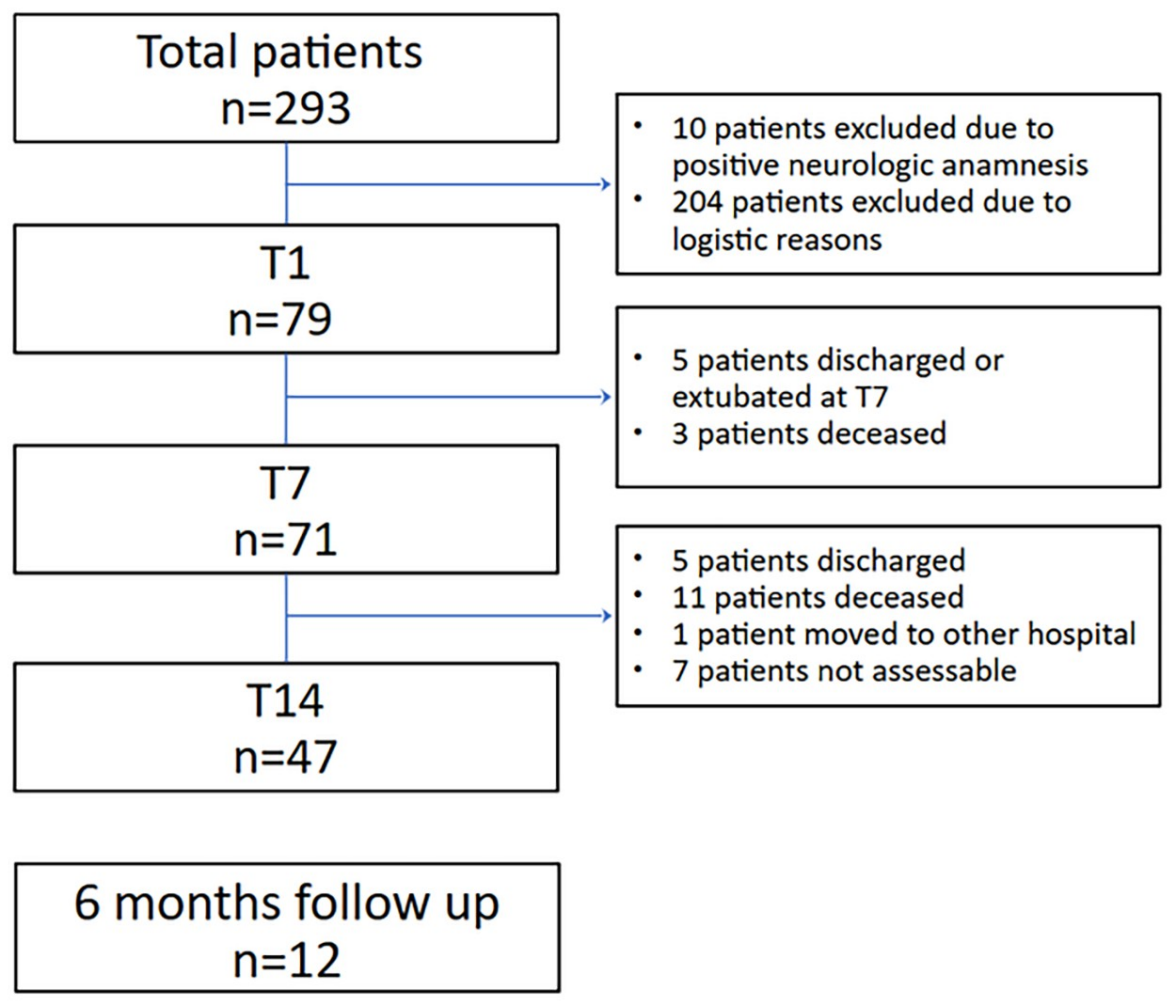
Enrollment flow-chart. Eighty patients were assessed on the day of admission. We included ICU admitted, mechanically ventilated patients because of severe respiratory failure COVID-19-related, confirmed by real-time polymerase chain reaction. We excluded patients under the age of 18, patients with pre-existing neurological diseases, and pregnant women.

Of 293 patients admitted from November 2020 to February 2021, 79 fulfilled the inclusion/exclusion criteria and were recruited at T1. In that period, the Alpha variant was the most widespread in Italy [21]. Seventy-one patients were examined at T7, and 47 at T14, on account of discharge or early deaths, as explained in Fig 1.

Table 1 summarizes the baseline characteristics. Patients were mostly male, with a median age of 65 (IQR 58–70). They had COVID-19-related severe respiratory failure (as shown by a median P/F ratio 131) and the level of respiratory support was similar to published reports [22]. Median ICU stay was 17 days (IQR 10–28); ICU mortality was 35%.

**Table 1:** **Panel A**: baseline characteristics, clinical parameters and outcome data- **Panel B**: time course of respiratory and hemodynamic parameters as indicators of disease severity and intensity of treatment.

**Panel A**			
**Baseline characteristics**		**Total population (n = 79)**	
Age (median [IQR]), yrs		65 [59;70]	
BMI (median [IQR])		28 [26;31]	
Males, no. (%)		61 (77)	
Comorbidities no. (%)			
None		21 (27)	
Arterial hypertension		51 (64)	
Cardiovascular disease		18 (23)	
Diabetes type 2		16 (20)	
COPD		5 (6)	
Other		20 (25)	
Inflammation at T1			
Lymphocytes (median [IQR])		583 [425; 925]	
Ferritin (median [IQR])		1278 [704;1806]	
C-Reactive protein		7.5 (4.1–15.3)	
D-dimer		1713 (733.8–4096)	
**Outcome**			
Length of stay (median [IQR])		16 [9–25]	
Days of ventilation (median [IQR])		17 [1–26]	
Deaths, no. (%)		27 (35)	
**Panel B**			
	**T1 (n = 79)**	**T7 (n = 71)**	**T14 (n = 47)**
**Mean airway pressure** (median [IQR]), cmH_2_O	17 [14–18]	15 [14–18]	14 [13–16]
Minute ventilation, (median [IQR]), L·min-1	8.0 [7.1–9]	8.0 [6.8–9.5]	8.3 [7.5–9.6]
**PEEP** (median [IQR]). cmH_2_O	11 [10–12]	12 [9–12]	8 [8–10]
FiO2% (median [IQR])	60 [50–80]	55 [40–70]	50 [40–65]
pH (median [IQR])	7.39 [7.36–7.44]	7.43 [7.35–7.47]	7.43 [7.37–7.46]
paO2 (median [IQR]). mmHg	80 [70–96]	79 [70–92]	85 [74–105]
paCO_2_ (median [IQR]). mmHg	49 [42–55]	57 [48–66]	50 [41–65]
P/F (median [IQR])	131 [93–180]	148 [113–193]	172 [120–242]
Hemodynamic catecholamine support. no. (%)	21 (27%)	13(18%)	10 (21%)
Average blood pressure (median [IQR]). mmHg	80 [72–90]	85 [73–99]	87 [74–97]
Central venous pressure (median [IQR]). mmHg	11 [8.13]	11 [9–13]	11 [8–14]
Sedation. no. (%)	75 (95%)	64 (90%)	42 (89%)
Neuromuscular blockade. no. (%)	68 (86%)	40 (63%)	20 (44%)

Continuous intravenous sedatives and opioids, often reinforced with myorelaxants, were necessary for most patients: 95% at T1, 90% at T7, and 89% at T14. The few patients who could be clinically examined had normal motor responses and preserved brain stem reflexes. The verbal response, impossible to explore in intubated patients, seemed preserved for the few receiving non-invasive ventilation.

### Pupils

Clinical examination detected no pupillary anomalies in size and light reactivity at admission and subsequent time points for all patients. These findings were confirmed by automated pupillometry in 13 patients (32 measurements): size and reactivity to light were normal across all time points. The pupil size, computed as the mean of the two eyes (expressed as median and IQR), was at T1 2.3 mm [2.0–2.5], at T7 2.3 mm [2.1–2.9] at T14 3.1 mm [2.7–5.3]. The Npi units also reported as median and IQR were normal in all cases: respectively 4.7 [4.5–4.7], 4.6 [4.2–4.8] and 4.3 [3.5–4.6] at T1. T7 and T14.

### Transcranial color doppler

**Fig 2:**
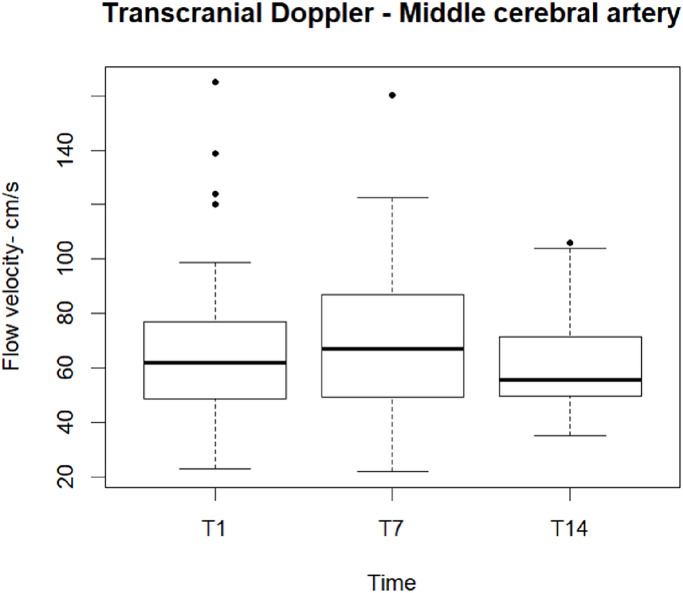
Middle cerebral artery mean flow velocity at three time points. Box and whisker plots showing mean flow velocity in the middle cerebral artery (at T1 n = 62, at T7 n = 58, at T14 n = 35). The lines in the boxes indicate the first quartile, median, and third quartile; the lower and upper bars indicate the minimum and the maximum values; black points indicate outliers.

A suitable acoustic window to visualize intracranial arteries was available in 68 patients. We performed 155 ultrasound (US) examinations: 62 at T1, 58 at T7, 35 at T14. The mean flow velocities were steady throughout the study: 62.18 cm/s [48.67–79.57] at T1, 67.00 cm/s [48.63–87.32] at T7, 55.60 cm/s [49.00–73.00] at T14. MCA mean flow velocity and flow pattern were within the range of normality [23] and remained stable throughout the study (Fig 2).

### Optic nerve sheath diameter measured with ultrasound

**Fig 3:**
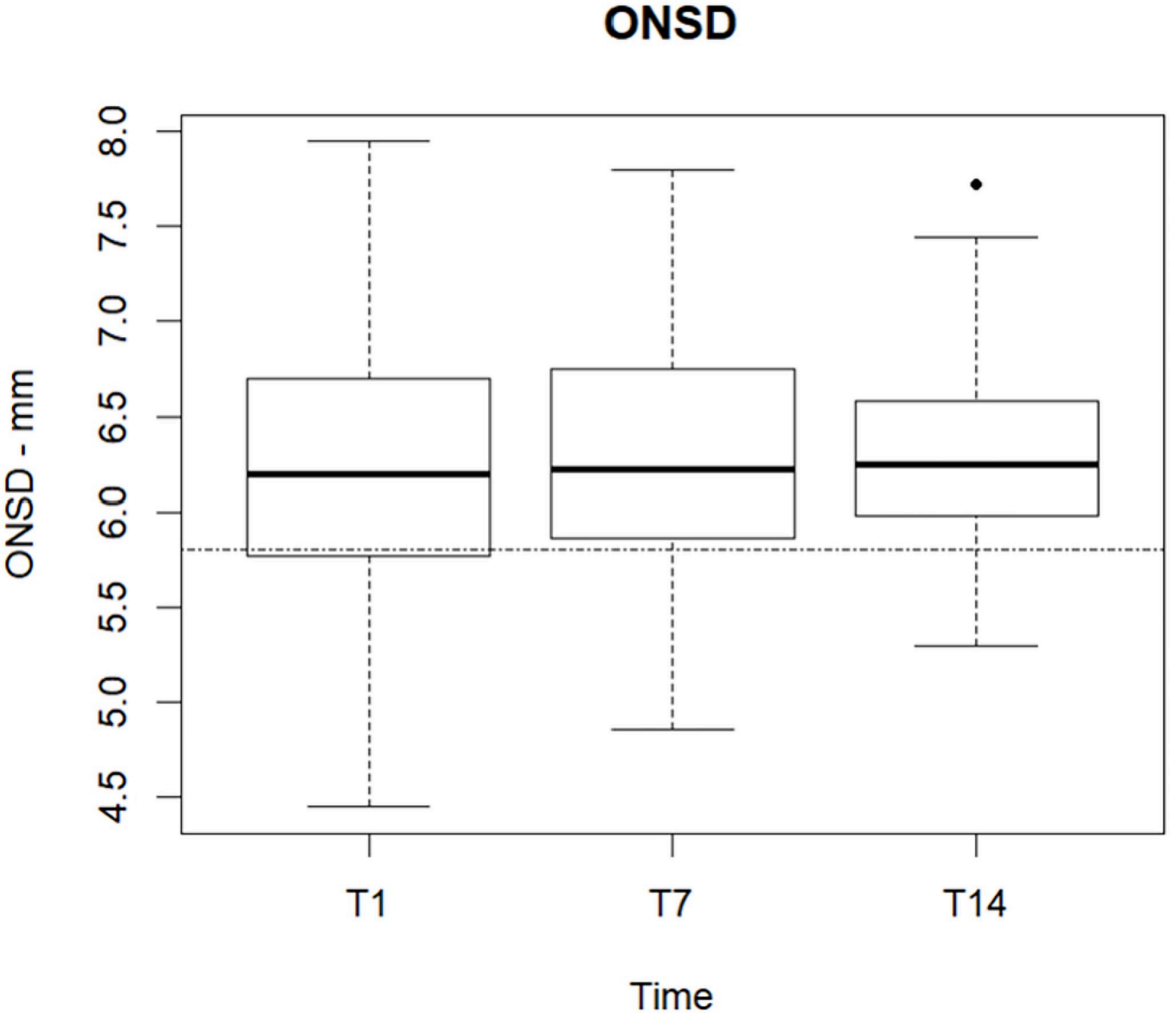
Optic Nerve Sheath Diameter (ONSD) in mm, measured by ultrasound. In this box-plot diagram, the horizontal line in each box represents the median, the limits of each box, the first and third quartiles, the whiskers, the maximum and minimum values, and the black points indicate the outliers. ONSD was measured in 69 patients at T1, in 66 patients at T7,in 40 patients at T14 and calculated as the mean of six bilateral measurements for each optic nerve. The dotted line refers to the diameter in the current literature, which seems unrelated to intracranial hypertension.

ONSD was measured with US in 69 patients, the results are summarized in Fig 3. Out of 175 measurements (69 at T1,66 at T7, 40 at T14), 138 (50 at T1, 53 at T7, 35 at T14) exceeded the threshold of 5.8 mm, considered in literature not related to intracranial hypertension [24].

**Fig 4:**
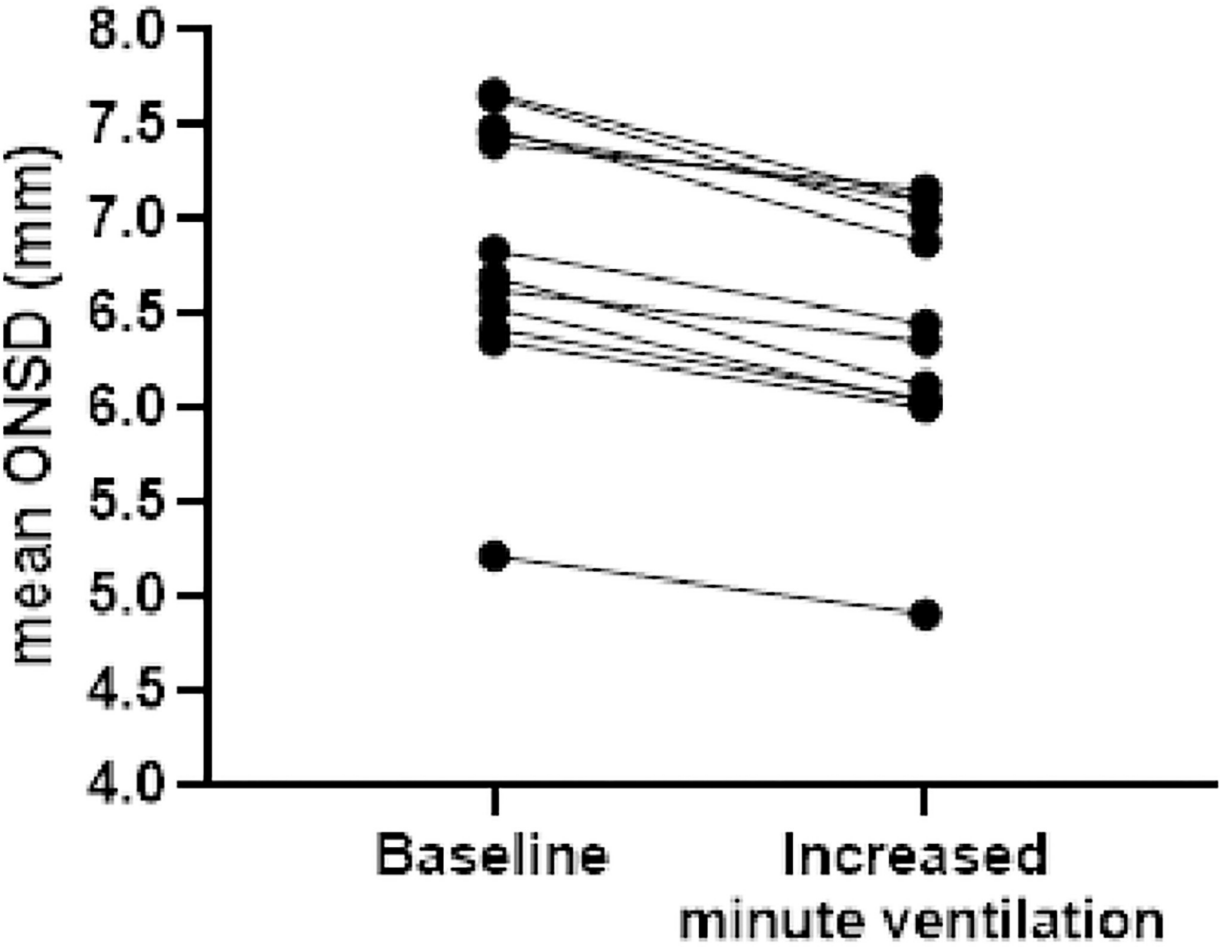
ONSD modifications induced by arterial paCO_2_ changes. In a subgroup of 12 patients, ONSD was measured at baseline and after an increase in minute ventilation.

In a subgroup of 12 patients, the ventilator setting was modified for clinical purposes, with an increase in minute ventilation and a reduction in PaCO_2_. The latter dropped from an initial median of 51 mmHg [49–59] to 45 mmHg [43–52] after the rise in minute ventilation (from 7.4 L/min [6.5–8.00] up to 9.7 L/min [8.8–10.4]). In these patients, when the US device was available during the changes, multiple ONSD measurements were taken before and after the ventilator setting was changed. Baseline ONSD was 6.76 mm [6.44–7.48] and decreased to 6.40 mm [6.03–7.08]. The difference was significant (p 0.0005) (Fig 4).

### Computed tomography

A total of 68 brain CT scans were done, 45 CT at T1 and 23 CT at T14. A mild subarachnoid hemorrhage in one patient and the hemorrhagic evolution of an ischemic stroke in another were detected on admission. Slight effacement of the cortical sulci was detected in two cases at T1 and two at T14. The basal cistern subarachnoid space was preserved in all patients.

**Fig 5:**
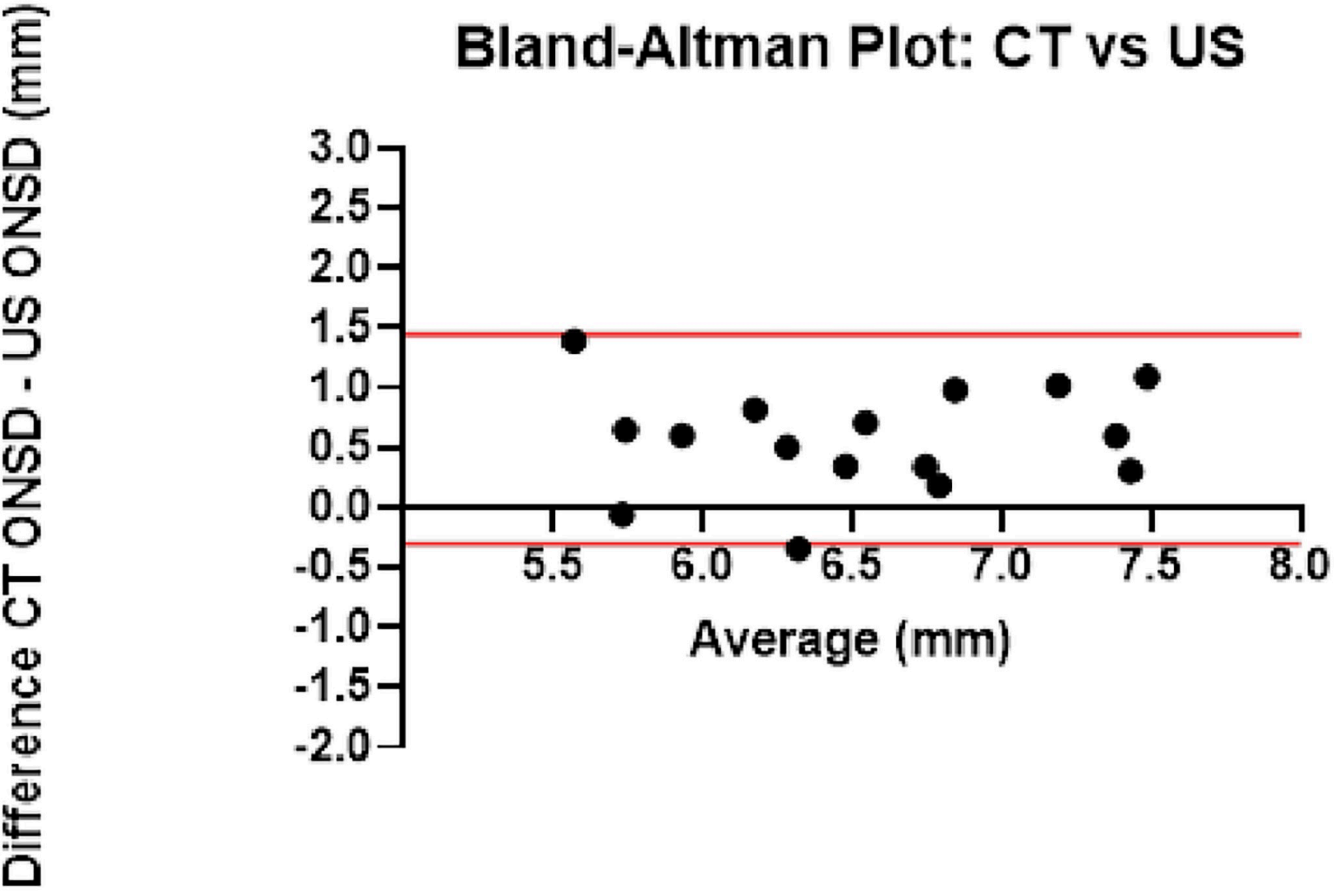
Bland-Altman plot indicating the agreement between the optic nerve sheath diameter measured with CT scan and US. The CT scan and the US examination were done on 16 patients on the same day. The Y axis shows the difference between the two measurements [(CT-ONSD)–(US-ONSD)], and the X axis presents their average. The limits of agreement (red lines) were -0.30 and 1.44 mm.

The ONSD in 68 CT scans was 6.90 mm [6.38–7.62] at T1 and 6.83 [5.99–7.30] at T14. In 16 patients, the ONSD was measured by US on the same day as the CT scan. This allowed a fair comparison of the ONSD indicated by the two techniques. The Bland-Altman analysis results are summarized in Fig 5. Agreement between the two methods was limited, with wide limits (-0.30–1.44 mm). Importantly, the ONSD measured from CT reconstructions were consistently higher than the US data.

### Outcome and follow-up

As indicated in Table 1, Panel A, 27 patients died in the ICU, and 52 were discharged alive to different units throughout the Lombardy Region. During the COVID-19 pandemic, the “Hospital in the Fair” served as a hub for more than 50 hospitals. This inevitably caused logistic problems for accurate centralized follow-up. We completed a follow-up examination with 12 patients at six months, during which we repeated a clinical neurological examination, measured the ONSD by US, and performed a venous blood gas analysis. Eighty-three percent of patients were males with a median age of 62 [53–67]; they reached the hospital by themselves or with the help of relatives, not requiring ambulance transport or medical assistance. No significant alterations were detectable at the neurologic examination (they were all awake, alert, and orientated, with no motor or sensitive deficits).

**Fig 6:**
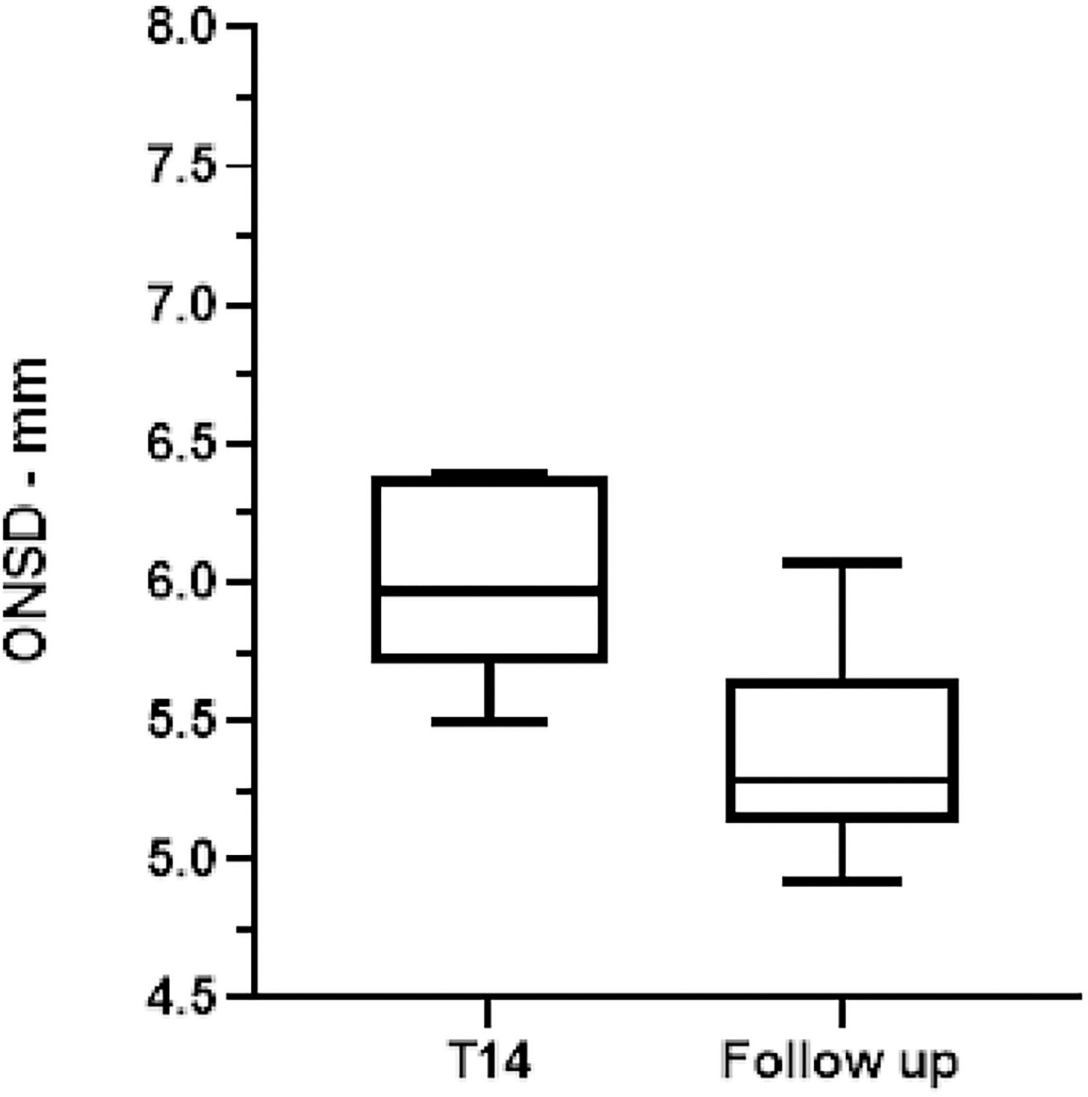
ONSD at T14 and six months after ICU discharge. Box and whisker plots showing ONSD at T14 and six months from ICU discharge in 12 patients. The lines indicate the first quartile, median, and third quartile; the lower and upper bars indicate the minimum and maximum values.

The US-ONSD had decreased in all patients, becoming normal in ten and only slightly elevated in two (Fig 6). The median ONSD six months after discharge was 5.28 mm [5.13-5-65]. The difference was significant when comparing the ONSD after the disease resolution with the last measure taken in the ICU (p <0.05). Peripheral venous blood gas analysis showed normal acid-base conditions (median pH 7.40 [7.35–7.41]), and the median pvCO2 was 43 mmHg [39–48]; the estimated paCO2 was normal (39.2 mmHg [35.2–41.2]). Only one patient had arterial pCO_2_ greater than 45 mmHg.

## Discussion

The study’s main aims were to investigate the potential neurological involvement of COVID-19 patients with severe respiratory failure and detect signs of increased intracranial pressure during the first 14 days in the ICU. Continuous infusion of sedatives and opioids, often reinforced with myorelaxants, precluded a reliable neurological evaluation in almost all patients. The few cases suitable for clinical examination had no alterations in their verbal and motor responses; brainstem reflexes appeared preserved. Pupil size and reactivity were explored in all patients, and no anomalies were detected either when the assessment was clinical or when the measurements were obtained by automated pupillometry. The median pupil diameter was less than 3 mm in the first two examinations, consistent with the generous use of opioids. Light reactivity, however, was preserved, and Npi was in the normal range. Importantly, pathological anisocoria was never detected. Repeated transcranial Doppler (TCCD) examinations showed normal mean flow velocity in the MCA in all cases. Of the 68 brain CT scans, only two showed pathological alterations (a mild subarachnoid hemorrhage and the hemorrhagic evolution of a previous ischemic stroke), not requiring treatment. All these findings in the normal ranges exclude any major involvement of the CNS and, in particular, exclude any major intracranial bleeding. This is reassuring, considering the rather strong anticoagulation protocol applied. However, our limited sample does not rule out the risk of intracerebral hemorrhage in COVID-19 patients, which seems rare but present. Data from a large registry (21,483 patients) identified an intracerebral hematoma in 48 cases (0.2%) [25]. Different data on clinical examinations and pupils were reported by the GECOVID-19 Group [26], which documented frequent neurological alterations, particularly delirium, and altered pupil reactivity in 31.03% of 29 cases. Important differences are likely between the two study populations and the clinical protocols applied and may at least partly explain the different findings. Delirium could not be assessed in our cases, given the depth of sedation applied, and pupils were explored with a different method (Neurolight Algiscan R, ID-MED, Marseille, France versus Neuroptics, Irvine, USA). Finally, our observation was limited to 14 days, while it was up to 33 days in the other study.

The second aim of this investigation, looking for hints of raised intracranial pressure, was based on brain CT scan analysis and repeated ONSD measurements. Two observers scored the CT scans considering the extent of sulcal effacement, peri-mesencephalic cistern effacement or disappearance, and loss of distinction between gray and white matter. According to previous reports [14–16], mainly regarding traumatic brain injury, these parameters seemed associated with intracranial hypertension. Despite a close search, our CT scans showed only a minor indicator (slight effacement of the cortical sulci) in four patients at two different time points. At the same time, the peri-mesencephalic cisterns and the distinction between gray and white matter were always normal. In contrast with these findings, which are close to normal, the ONSD was pathologically enlarged: 79% of measurements at various time points exceeded the threshold associated with intracranial hypertension. We also measured the ONSD in 68 CT scans to confirm this, where pathologically wide diameters (median 6.38 mm) were identified. Previous reports have documented the correlation between diameters measured on the CT scan and actual ICP measured invasively [27]. A Bland-Altman analysis in 16 cases where the CT scan was acquired the same day as the US measurement confirmed pathologically wide diameters with both techniques. Despite the broad limits of agreement between these two methods, the CT scans excluded any overestimation by the US technique. Judging from this data, an ONSD enlargement in our patients seems well documented, with values consistent with intracranial hypertension. This is in agreement with data provided by the GECOVID-19 Group [26].

Intracranial hypertension would require, besides the identification of the cause (infection, hemorrhage, ischemic stroke, etc.), an invasive measurement to confirm the suspect and subsequently the application of neuroprotective strategies (i.e., providing normothermia, adequate sedation, monitoring of ICP, proper ventilation and control of airway pressure, appropriate hemodynamics and cerebral perfusion and regulation of electrolytes and glucose). Due to the anti-coagulation treatment, we could not perform any invasive measurements on our population to support our hypothesis. The intensity of treatment for intracranial hypertension can be wide, and some aspects collide with the clinical management of COVID-19 respiratory failure, which was the reason our patients were admitted and treated in the ICU. Permissive hypercapnia, for instance, is incompatible with intracranial hypertension treatment because of its effects on the cerebral blood flow.

Multiple factors could have contributed to our hypothesis that the ONSD enlargement was due to raised ICP.

Hypercapnia, which causes cerebral vasodilation [28], may play a role, together with the effects of mechanical ventilation. In particular, positive airway pressure, by raising intrathoracic pressures and preventing venous return from the brain, may affect ICP, as seen in animal models [29] and in human clinical studies in the setting of acute brain injury [30]. Two facts support hypercapnia as a potential explanation for the ONSD enlargement. When it was possible to explore acute paCO2 reductions in a subset of patients, those reductions were associated, at constant PEEP levels, with concomitant ONSD decreases. Accordingly, when some patients regained normocapnia (as estimated by venous gas analysis six months after discharge), their ONSD went back to normal. These data do suggest a prominent role of hypercapnia in the ONSD enlargement as an indicator of increased ICP that could be related to intracerebral vasodilation. Although our cohort presented a moderate respiratory failure, hypoxia could have participated to the rise of intracranial pressure, working together with hypercapnia as a stimulus to increase cerebral blood flow (CBF) and vasodilation. We should also consider that the CNS could be involved in the COVID-19 infection both directly, since the known neurotropism of SARS-CoV-2, and indirectly, as a consequence of the systemic inflammation and cytokin cytokine storms. In details, viral neuro-invasion could follow several routes [31]. The wide expression of the ACE-2 receptor in neurons, glia, and vessels makes them vulnerable to SARS-CoV-2 infection. Given the key role of immune response in the pathogenesis of SARS-CoV-2 infection [32], enlarged ONSD could also be an expression of the systemic inflammation: inflammatory mediators could lead to the alteration of brain blood barrier permeability and contributing contribute not only to neuro-invasion but also to a break of the homeostasis [33]. In this perspective the demyelinating syndromes [34] and the neuro-ophtalmic inflammatory manifestations [35] described in association with COVID-19 must be introduced. Both optic neuritis (ON) and optic perineuritis (OPN), in which inflammation affects respectively the optic nerve axons and the optic nerve sheath, cause the increase of the ONSD [36, 37]. Since our cohort did not experience any symptoms or signs related to ON or OPN, we ruled out these hypothesis: no one reported any vision alterations and pupils were normal; the fundoscopy was physiologic in two patients who underwent ophtalomogic assessment because of the development of severe conjunctivitis.

### Limitations

Our study has obvious limitations: the need for continuous sedation and, in some cases, for neuromuscular blockade, which prevented a complete neurological examination and concealed potential acute changes. The number of patients examined, particularly at follow-up, was very limited. We could not rely on invasive measurements to confirm our suspicion of intracranial hypertension, which remains linked to an indirect sign (ONSD). Finally, considering only the limited observation period, we could not exclude later neurological complications during a longer ICU stay.

## Conclusion

In a cohort of COVID-19 patients admitted to an ICU for acute respiratory failure and examined by a bundle of non-invasive measurements, the most altered parameter was ONSD enlargement, consistent with raised ICP. This seemed dynamically related to hypercapnia and resolved at follow-up. Further studies now seem justified, exploring the ONSD behavior in mechanically ventilated patients with severe respiratory failure and hypercapnia.

## Abbreviations

CNS: Central nervous system
CO_2_: Carbon dioxide
COVID-19: Coronavirus disease
CT: Computed Tomography
CT-ONSD: Optic nerve sheath diameter measured by computed tomography method
ICP: Intracranial pressure
ICU: Intensive Care Unit
IQR: Interquartile range
LOS: Length of stay
MCA: Middle cerebral artery
NPi: Neurological pupillary index ^®^
ON: Optic Neuritis
ONSD: Optic nerve sheath diameter
OPN: Optic Perineuritis
paCO2: Arterial partial pressure of carbon dioxide
PEEP: Positive end-expiratory pressure
SARS-CoV-2: Severe acute respiratory syndrome coronavirus 2
T1: Time 1, corresponding to the admission day
T14: Time 14, corresponding to the fourteenth day of hospitalization
T7: Time 7, corresponding to the seventh day of hospitalization
TCCD: Transcranial color Doppler
US: Ultrasound
US-ONSD: Optic nerve sheath diameter measured by ultrasound method
