# Correction: Suspected intracranial hypertension in COVID-19 patients with severe respiratory failure

**DOI:** 10.1371/journal.pone.0313211

**Published:** 2024-10-30

**Authors:** Marco Carbonara, Erica Ferrari, Tatiana Birg, Veronica Punzi, Francesca Bichi, Beatrice Lazzari, Valentina Palmaverdi, Nicola Bottino, Fabrizio Ortolano, Tommaso Zoerle, Giorgio Conte, Nino Stocchetti, Elisa R. Zanier, The UOC Terapia Intensiva Presidio Temporaneo Fiera Milano Investigators group

The published funding statement is incorrect. The correct funding statement is as follows: This study was partially funded by the Italian Ministry of Health under the current research program of IRCCS (Istituti di Ricovero e Cura a Carattere Scientifico).
